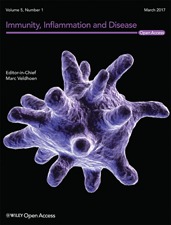# Issue Information

**DOI:** 10.1002/iid3.128

**Published:** 2017-02-23

## Abstract